# The Missing Piece in Chronic Disease Prevention: Dental Caries

**DOI:** 10.5888/pcd22.250130

**Published:** 2025-08-07

**Authors:** Kiran P. Nagdeo

**Affiliations:** 1 Department of Epidemiology, NYU School of Global Public Health, New York

Chronic diseases such as cardiovascular disease, diabetes, and cancer receive significant attention in global public health agendas (1). Billions of dollars are invested annually in the research, prevention, and treatment for these diseases. Yet, the most prevalent chronic disease globally — dental caries — is largely neglected in these conversations (2,3). Oral diseases affect an estimated 3.7 billion people worldwide, with dental caries accounting for most cases (4). Caries often begins in early childhood and continues throughout life, silently affecting billions of people. Despite its overwhelming prevalence, oral health is frequently excluded from broader public health strategies, perpetuating cycles of inequality, especially in low-resource settings (3).

Dental caries is a preventable condition, yet it disproportionately affects people that have been socially and economically marginalized (5). In many communities, systemic barriers such as inadequate access to care and a lack of oral health education persist (5). These barriers are deeply intertwined with social determinants of health. People with low incomes and limited formal education or who reside in rural areas often have little to no access to basic preventive services (6). In high-income countries, disparities emerge through delayed treatment and cost-related avoidance of care. In low- and middle-income countries, entire populations may lack access to professional dental services altogether (6). The gap in access to preventive and restorative oral health care remains one of the clearest indicators of global health inequality (5,6).

Despite overwhelming evidence to the contrary, the public health community has long viewed oral health as a separate, siloed issue, distinct from systemic health (7). Untreated dental caries is not only painful and disabling but also affects school attendance, employment, and quality of life (8,9). It is also associated with such chronic illnesses as cardiovascular disease (10) and diabetes (11), and its sequelae can lead to adverse pregnancy outcomes (12–14). If left untreated, dental caries can progress to severe odontogenic infections, which may become life-threatening without timely care (15). The exclusion of oral health from universal health coverage schemes and national health priorities reinforces this crisis (6). Furthermore, the cost of treatment is often prohibitive, which exacerbates the poverty that some families experience (16). Global estimates suggest that the economic impact of oral diseases, including dental caries, totals hundreds of billions of dollars annually in direct and indirect costs, resulting in a silent economic burden that public health systems are ill equipped to manage (16).

What makes dental caries a particularly frustrating public health issue is that it is easily preventable with simple, cost-effective interventions (17,18). Fluoride, whether through toothpaste, water fluoridation, or varnishes, is an inexpensive and powerful preventive tool (18). Yet billions of people live in areas without access to fluoridated water, leaving them unprotected against caries (19). Meanwhile, sugar consumption, a key risk factor for caries, is often unregulated, especially in low- and middle-income countries where sugary products are aggressively marketed (20). Public health systems have yet to address these structural drivers through coordinated action. While addressing caries requires individual behavior change, it also demands upstream policy interventions to limit corporate influence, regulate food labeling, and create environments that support oral health. Without policies that restrict advertising to children, subsidize healthier food alternatives, and improve access to fluoride, even the most well-meaning educational campaigns may fall short (20).

Current responses to dental caries are insufficient and fragmented. Public health tools such as community water fluoridation, school-based sealant programs, and nonprofit mobile clinics are effective but remain underfunded or inconsistently implemented (21). These approaches treat oral health as a peripheral issue, and their reliance on short-term funding undermines sustainability. The absence of systemic integration and innovative policy frameworks hampers progress and leaves billions of people underserved. Additionally, national health strategies rarely incorporate oral health targets or metrics, limiting accountability and visibility within broader health system reforms (6). 

Some countries have successfully integrated dental care into their national universal health coverage programs. For example, Brazil’s *Programa Brasil Sorridente* (Smiling Brazil Program) has expanded community-based oral health teams as part of the national health system, improving preventive service coverage and reducing untreated caries (22,23). Similarly, Thailand’s UHC model includes essential dental services delivered through primary care networks (24,25). In contrast, countries without such integration, such as the US, continue to face severe access gaps, especially among low-income populations who are often uninsured for dental services (6,26). A lack of consistent surveillance data further hinders efforts to assess program effectiveness and adjust strategies (6). Many countries do not conduct regular national oral health surveys, and where data exist, they often exclude key indicators such as untreated caries or barriers to care (6).

 Addressing workforce shortages is equally critical. Policymakers should support the training and licensing of mid-level providers, such as dental therapists, to deliver preventive and basic restorative services, especially in rural and underserved areas. Successful models from countries like New Zealand and Canada demonstrate the potential of task shifting to close gaps in access to service (27). Additionally, integrating oral health screenings into routine medical visits, such as child immunizations and maternal health appointments, can ensure that caries prevention is embedded across the life course (6). This life-course approach not only improves outcomes but also promotes efficiency and continuity within health systems. Interprofessional education should be expanded to ensure that doctors, nurses, and community health workers understand their role in supporting oral health, creating a more collaborative and cohesive health care ecosystem.

Behavioral and cultural innovation must accompany policy reform. Oral health education must be tailored to the social and cultural contexts of the communities it serves. Community-based approaches that use trusted local leaders, traditional storytelling, and culturally relevant messaging can empower people to adopt preventive behaviors (28,29). Behavioral nudges, such as mobile telephone reminders, school competitions, and peer-led programs, can encourage healthy habits from a young age (30,31). Schools, religious institutions, and youth clubs all offer untapped potential as oral health promotion partners. Public health campaigns should also be co-designed with community input to ensure that messages resonate and materials are accessible. Sustainability should also be embedded in these interventions. Green dentistry initiatives, such as biodegradable dental products and solar-powered mobile clinics, can deliver care in climate-vulnerable regions while minimizing environmental harm. As the climate crisis grows, so does the need for resilient, sustainable oral health infrastructure that is environmentally responsible and adaptable to local contexts.

Despite the promise of these interventions, several implementation challenges persist. In many low- and middle-income countries, funding limitations, shortages of trained personnel, and weak health infrastructure undermine the sustainability of oral health programs (32). Policy reforms often face resistance due to competing priorities or a lack of political will (33,34). Furthermore, behavioral interventions may falter without culturally tailored approaches or long-term community engagement (29). To ensure effectiveness, oral health initiatives must be context-specific, embedded within primary care, and supported by integrated financing and monitoring mechanisms. The benefits of these interventions would be wide-reaching. Children in low-income communities would experience fewer missed school days due to pain (8,9). Rural and climate-affected populations would gain consistent access to essential care. Aging adults could maintain dignity and quality of life without chronic dental pain. Health systems would reduce expenditures on emergency dental treatments and preventable complications (16). Most importantly, global development efforts would be strengthened by advancing oral health equity. By investing in caries prevention, we also invest in better nutrition, educational attainment, maternal outcomes, and workforce productivity across generations. Tackling dental caries is not just about teeth; it is about justice, opportunity, and unlocking human potential. Oral health equity is not a luxury — it is a prerequisite for dignity, well-being, and public health justice.
